# Vindiciæ Medicæ; or, a Defence of the College of Physicians

**Published:** 1834-07-01

**Authors:** 


### Vindiciæ Medicæ; or a Defence of the College of Physicians. By *Sir George Tuthill*, M.D. Fellow of the College.

A more feeble vindication of an obsolete, but tyrannical monopoly was never sent forth into the world. Nine-tenths of the pamphlet are occupied with old musty records and decisions, to prove the *legality* of laws that outrage common sense—and that in the year 1834 !! There is a halo of prejudice and pride surrounding every corporation—and especially the College of Physicians, which completely distorts the vision of those within that halo—at least many of them—for, happily, a respectable minority of the Fellows of the College see clearly the imperfections of their institution. It would be as impossible for the old Fellows to change their opinions and cast off their prejudices, as for an Ethiopian to change his skin and become white. It is totally useless to argue with them. They are as blind to the signs of the times as a mole to the phases of the moon! Not so with a great many of the young Fellows. Their brains are not so hardened by age as to resist every impression of liberality—the avenues to light and knowledge are not blocked up—and they see the state of things around them. They are aware that time alone, without any Parliamentary interference, would gradually, aye and quickly too, break down the unnatural and galling barriers that separate the members of one common (and oh! that we could say a liberal) science! They are not insensible to all feelings but those of selfishness and pride—and hence the contrast of their evidence, before the parliamentary committee, with that of their seniors.

We will not spend our time so unprofitably as the author has done, in going over all the old, vapid, and perhaps *unjust* decisions of tory and high prerogative judges. Let them *sleep* in the musty archives of the College. That the College and its advocates are now driven to the necessity of resting all their pre-eminence and superiority on the high tone of *morality*, and the deep tincture of *religion* which they have imbibed at Oxford and Cambridge, will be evident from the following extract.

"The College never contemplates the acquisition of *medical knowledge* either at Cambridge or Oxford; but it does contemplate the acquisition of that intellectual and *moral* condition which is best adapted to the pursuit of philosophical researches, and to the *religious observance of all social duties.* The successful practice of physic perpetually involves a just and rapid induction from the simultaneous or concurrent existence of great numbers of minute facts, that can only be appreciated by a mind trained to minute research, to logical induction, and to mathematical exactness. It is this mental training which the studies of Cambridge and of Oxford are intended to secure.

The social relations under which the Physician is placed, the delicate and complicated conditions under which he is perpetually invited to offer some relief to human misery, render it equally imperative that the highest sense of honour and probity should be demanded and insured. Of this *religious and moral training*, the discipline of the *English* Universities has afforded the best security." 83.

Holy St. Francis! is it come to this? Have the *medical* Fellows of Oxford and Cambridge monopolized all the morality and religion of the profession, as well as all the snug appointments belonging to the Corporation! Must we poor licentiates be damned in the next world for deficiency of religion—while we are degraded in this world, for want of that morality which is concentrated in Oxford and Cambridge! Really, this decision does not savour much of Christian charity in our judges; and we apprehend that it will require better arguments than any which we see in this pamphlet, to persuade the world that the congregations of youth, in our English universities, are circumstances very particularly calculated to produce a superabundance of either morality or religion, beyond what is acquired in Edinburgh or Glasgow. On the contrary, we unhesitatingly maintain that, from no two points in England, Ireland, or Scotland, of equal extent or population, does such a mass of vice, immorality, and irreligion, radiate annually upon society, as from Oxford and Cambridge. If Sir George Tuthill does not know this (and in charity we hope he does not), then he knows very little of the world, of human nature—or of the English universities.

We are sorry to say that, throughout the whole of this Vindication, there prevails a spirit of quibbling sophistry, misrepresentation, and special pleading, that does little honour to a member of any learned profession—and will do no good to the rotten cause which the brochure is intended to support.

It is with reluctance we advert to specimens; but we are bound to shew some proof of the justice of our censures.

Thus, to the complaint of the Licentiates, that the "Fellows have usurped all the corporate power, offices, privileges, and emoluments attached to the College," the following *candid* answer is given.

"It is true that a Physician, licensed to practise in London, is 'excluded from the offices of the College, and from any share in the management of the corporation,' so long as he remains in the order of licentiates; *but he does not necessarily remain in that order. Licentiates are elected into the Fellowship, and become thereby eligible to every office which the College contains.* There is, therefore, no exclusion."

Now was there ever such a jesuitical, disingenuous answer as this given by a man, who boasts of having imbibed that high tone of morality, and that sublime effusion of religion, which Oxford and Cambridge alone can supply? Because a toad-eater, or Sir Pertinax Mac Sycophant of the College is, once in every year or two, admitted to the Fellowship, the pamphleteer unblushingly tells us that "there is no exclusion of the licentiates"!! Proh pudor!

The above is a fair specimen of the quibbling sophistry which pervades the vindication—and which to use the author's own words (p. 74), is "an auxiliary that honesty and virtue never employ."

Now for a specimen of misrepresentation. "The *successful* licentiates have not admitted that they are subjected to any practical disadvantage." This is *not fact.* Let any one look at the list of malcontent licentiates who have signed the petition to Parliament, and he will acknowledge that there are *many* men there who have been eminently *successful,* in every sense of the word—perhaps more successful than the titled FELLOW who makes this insulting and unfounded assertion. But, whether the successful Licentiates admit or not the existence of "practical disadvantage," we assert, in direct contradiction to the author, that it does exist, and to a vast extent, as every Fellow of the College well knows. Whenever the interests of the Fellow and the Licentiate clash, whether in private practice or in competition for public situations, the Licentiate very generally receives damage. Every engine is immediately set to work by the friends of the Fellows, and sometimes, we grieve to say, by some of the Fellows themselves, to represent the Licentiate as being of a lower caste—as not, in fact, being a regular physician at all—but only, as they jesuitically say, a " LICENTIATE"—a person *licensed* to practise in simple cases, and among inferior classes of society. Hundreds of instances of this kind have come to our knowledge. So notorious is this species of medical tactics in the metropolis, that many Licentiates of talent and experience are deterred from becoming candidates for hospitals, &c. lest their professional character should be injured or destroyed by the use which can be made of this odious distinction ! Hence the anxiety of the author of this pamphlet, and others of his politics, to keep up the difference of grade among metropolitan physicians.

We shall conclude these strictures, by exposing one of the most ludicrous absurdities that ever entered into the ratiocination of any man, not confined in Bedlam.

The petitioners submitted that the College of Physicians, as at present constituted, was wholly inadequate to the due regulation of the medical profession in this country—and, further, that it did not provide for the practice of physicians in the provincial towns. Let us hear the sapient and *sane* answer.

ANSWER.—" The first member of this allegation is proved to be false by a single fact ; namely, that the Medical Profession is more exalted in this country than in any other country of Europe.

The second accusation which this paragraph of the Petition involves, is the want of that protection which should be afforded to the public by the College against unskilful practitioners. *But the public do not complain : and the capital of England is the healthiest capital in Europe.*"

Shade of Locke ! or of any other man who ever wrote upon, or possessed, a " human understanding," was there ever such reasoning as this ushered into the world—and that, too, by an Oxonian ? Because the medical profession in England (over which the College has no control beyond the seventh mile-stone—and only over the Fellows and Licentiates within that range) is more respectable than in other countries, *ergo*, the College of Physicians is the cause of this superiority ! But the climax of absurdity is not yet gained. Because the public do not complain—and because London is the healthiest capital in the world, *ergo*, the College of Physicians affords ample protection to the public against quacks and unskilful practitioners !!!*

We drop the pen in pity and contempt for such wretched drivellings—such perversions of common understanding, that were they exhibited by any poor FELLOW in GRAY'S-INN COFFEE-HOUSE, they would consign him, without further proof, to the dreary cell of a madhouse for the remainder of his life !

We will offer no excuse—we scorn it—for the severity of these censures. The author of the pamphlet has used all his efforts to represent, directly or

---

* The legion of quacks might shew a much better argument on their side. They might urge, that the cause of London longevity is to be attributed to the want of power, on the part of the College, to interrupt the salutary operations of the charlatan host.

by implication, the petitioning Licentiates as a body of discontented, unsuccessful, prevaricating, dishonest, nay, *lying* practitioners, who have ungratefully rebelled against the most pure, moral, religious, and useful corporation that ever existed on the face of the earth. Let the labourer, therefore, receive his reward. We have here exhibited the true character of his lucubrations and vindications, from the rising to the setting sun—from the springs of the Ganges to the sources of the Ohio.

